# Advice to Those Who Wear Artificial Teeth

**Published:** 1886-07

**Authors:** 


ARTICLE VII.

### ADVICE TO THOSE WHO WEAR ARTIFICIAL TEETH.

The varied scenes and trials through which every one must pass before artificial teeth are necessary, are not easily forgotten. They remenber how (in many cases by neglect) one tooth after another decayed, till the nerve became exposed and ulcers formed, producing the most excruciating pain; but now the last offending member has been extracted, and "my troubles are ended. I can now have artificial teeth in every way as good as natural ones." To correct some erroneous opinions on this point is the object of this article.

Artificial teeth, properly made, will answer many purposes of natural teeth, but no dentist can insert teeth which will answer those purposes as *well* as natural ones.

There are many difficulties attending the wearing of artificial dentures which, in the main, by patience and perseverance, may be overcome.

1st. The presence of the plate in the mouth at first, specially when the patient has been without teeth for a long time, is a source of inconvenience. A few days of patient use will remove this trouble.

2d. Many complain of the plate chafing the gums, producing soreness. This difficulty comes mainly when the plate is inserted soon after the teeth are extracted; the gum heals over the sharp, bony points of the sockets; the plate pressing on the gums causes these points to cut through to the plate; in a few days these points will absorb, the gum heal, and the plate will be worn with ease. If the edge of the plate cuts into the contiguous muscle, of course, this edge should be cut back.

3rd. Others complain that the plate produces an unpleasant taste in the mouth. This may be because it is made of base material; when good material is used, such as continuous gum, gold, or vulcanite, this difficulty will not exist, if the plate is kept clean. When eating, fine, starchy particles of food will adhere to the plate; if not removed, it will soon sour, producing an unpleasant taste. The plate should be cleansed after each meal.

4th. The difficulty most complained of, specially in full sets (and *partial* sets where clasps are discarded) is the inability to use the teeth, when first inserted. This difficulty occurs in every case to some degree, and to overcome it, much depends on the patience, perseverance, and aptness of the wearer. To be more explicit, the upper plate is held up by suction, with a force varying from eight to fifteen pounds. The main object of this suction is to keep the plate from dropping when speaking, laughing or eating.

The teeth are required to be set on the plate at an angle of from ten to twenty degrees. The force of an ordinary bite is about fifty pounds, which, if applied to the front teeth at this angle, in the same manner in which we would bite with the natural teeth, would, of course, overcome the eight or fifteen pounds atmospheric pressure, causing the plate to tip. The same is true in chewing on one side of the mouth. To remedy this difficulty, it is necessary for the patient to learn to press the food against the front teeth at the same time they are brought together, and at first to learn to chew on both sides. To so learn this process, till it becomes a habit, usually requires some time.—*Allport's Dental Journal.*

---

ARTICLE VIII.

